# Diagnostic accuracy of MRI for detecting nerve injury in brachial plexus birth injury

**DOI:** 10.1093/bjr/tqae214

**Published:** 2024-10-21

**Authors:** James Brooks, Claire Hardie, Ryckie Wade, Irvin Teh, Grainne Bourke

**Affiliations:** Leeds Institute for Medical Research, University of Leeds, Leeds, LS2 9JT, United Kingdom; Leeds Institute for Medical Research, University of Leeds, Leeds, LS2 9JT, United Kingdom; Department of Plastic and Reconstructive Surgery, Leeds Teaching Hospitals Trust, Leeds, LS1 3EX, United Kingdom; Leeds Institute for Medical Research, University of Leeds, Leeds, LS2 9JT, United Kingdom; Department of Plastic and Reconstructive Surgery, Leeds Teaching Hospitals Trust, Leeds, LS1 3EX, United Kingdom; Leeds Institute of Cardiovascular and Metabolic Medicine, University of Leeds, Leeds, LS2 9JT, United Kingdom; Leeds Institute for Medical Research, University of Leeds, Leeds, LS2 9JT, United Kingdom; Department of Plastic and Reconstructive Surgery, Leeds Teaching Hospitals Trust, Leeds, LS1 3EX, United Kingdom; Department of Integrative Medical Biology, University of Umea, Umea, SE-901 87, Sweden

**Keywords:** MRI, brachial plexus injury, obstetric

## Abstract

**Objectives:**

To determine the diagnostic accuracy of MRI for diagnosing nerve injury in brachial plexus birth injury (BPBI).

**Methods:**

Electronic databases were searched from inception to February 15, 2023 for studies reporting the accuracy of MRI (index test) compared to surgical exploration (reference standard) in detecting the target conditions of: root avulsion, any nerve abnormality, and pseudomeningocele (as a marker of root avulsion) in children with BPBI. Meta-analysis using a bivariate model was performed where data allowed.

**Results:**

Eight studies met the inclusion criteria. In total, 116 children with BPBI were included. All included studies were at risk of bias. The mean sensitivity and mean specificity of MRI for detecting root avulsion was 68% (95% CI: 55%, 79%) and 89% (95% CI: 78%, 95%), respectively. Pseudomeningocele was not a reliable marker of avulsion. Data were too sparse to determine the diagnostic accuracy of MRI for any nerve abnormality.

**Conclusions:**

At present, surgical exploration should remain as the diagnostic modality of choice for BPBI due to the modest diagnostic accuracy of MRI in detecting root avulsion. The diagnostic accuracy of MRI needs to be close to 100% as the results may determine whether a child undergoes invasive surgery.

**Advances in knowledge:**

Previous research regarding MRI in detecting BPBI is highly variable and prior to our study the overall diagnostic accuracy was unclear. Through conducting a systematic review and meta-analysis, we were able to reliably determine the overall sensitivity and specificity of MRI for detecting root avulsion.

## Introduction

Brachial plexus birth injury (BPBI) affects 0.4-2 children per 1000 births^1^ and is often the result of traction to the neck during complicated childbirth. Roots C5 and C6 are most often damaged (Erb’s palsy) however nerve roots C5-T1 can be involved.^2^ Within the first 3 months of life spontaneous recovery is common, however in 10-30% recovery is incomplete^3^ which can lead to lifelong issues such as joint deformity, loss of function,^4^^,^^5^ and psychological morbidity.^6^

Functional outcomes depend on the severity of nerve damage. Neuropraxia (stretching of the nerves) is associated with the best prognosis. Nerve rupture and root avulsion (nerve roots are separated from the spinal cord entirely) both result in permanent injury and require nerve reconstruction if any function is to be regained.^7^ Determining the extent of injury and reliably predicting the long-term sequela remains a challenge. Surgical exploration to visualise the brachial plexus can be undertaken if there is inadequate clinical recovery and damaged nerves reconstructed with nerve grafts or transfers. Surgical exploration of the plexus is typically supported with use of somatosensory evoked potentials and bipolar nerve stimulation. Whilst this approach is diagnostically the “gold standard”, there are risks associated with operating on infants, including accidental extubation, postoperative fluid overload, and phrenic nerve injury.^8^

Imaging modalities such as MRI offer a non-invasive method of visualising the brachial plexus. The reported diagnostic accuracy of MRI in detecting BPBI is variable.^9–12^ MRI is particularly advantageous given the absence of ionising radiation and intrathecal contrast, however anaesthetic risks still apply. The ability to differentiate between pre- and post-ganglionic injury is vital because they require different reconstruction and confer different prognoses. Pre-ganglionic injury confers the worst prognosis and requires treatment with nerve transfer because the native motor neurons recede.^13^ Post-ganglionic injury is typically associated with better outcomes and motor function can be recovered if nerve continuity is surgically restored.^14^^,^^15^ Extradural accumulation of cerebrospinal fluid can form pseudomeningoceles which are considered a surrogate marker of root avulsion, however the accuracy of this is demonstrably poor in adults^16^ and remains unclear in children.

An imaging modality that is diagnostically accurate could potentially facilitate earlier treatment and better functional outcomes, as well as aid long-term predictions of function. Furthermore, imaging can be performed prior to 12 weeks which could facilitate earlier treatment and better functional outcomes.^17^ This review aims to clarify the current diagnostic accuracy of morphological MRI for detecting nerve injury with the intention to define its role clinically and highlight areas of future development.

## Methods

This systematic review and meta-analysis followed the Preferred Reporting Items for Systematic Reviews and Meta-analyses-Diagnostic Test Accuracy (PRIMSA-DTA) guidelines^18^ and was constructed using the Cochrane Diagnostic Test Accuracy Protocol.^19^ This systematic review was registered prospectively on PROSPERO (CRD42021267629) and conducted in line with our protocol.^20^

### Studies and participants

Studies that reported the diagnostic accuracy of pre-operative MRI in comparison to surgical exploration were included. This review involved infants under two years old with BPBI, regardless of gender, ethnicity, or disease severity. Children with bilateral injuries were also included. Case reports and review articles were excluded, all other study types were eligible.

### Target condition

The primary target condition was root avulsion of the brachial plexus. Secondary target conditions were pseudomeningocele and any nerve abnormality (to include avulsion, pseudomeningocele, or post-ganglionic injury, eg, neuroma, scarring, or oedema of the nerve). The ability of MRI to distinguish between normal roots and any number of root avulsions was investigated.

### Index test

The primary role of MRI is to identify the morphology (presence vs absence) of root avulsion. A lack of continuity between the spinal cord and spinal root at the level of the exit foramen is regarded as a positive finding for avulsion. Roots C4-T2 can be affected and bilateral injury can occur in rare cases. Clinically, the presence of a single root avulsion is of equal importance to any number of avulsions given that both cases warrant reconstruction by nerve transfer. MRI is also capable of detecting pseudomeningoceles which appear as an abnormal contour of the dura and collection of dorsal extraspinous fluid.^21^ Pseudomeningoceles are considered a surrogate marker of avulsion because their formation involves rupture of the dura mater which can imply the nerve root is also ruptured. However, the sensitivity and specificity of pseudomeningocele as a surrogate marker of avulsion is unclear^22^^,^^23^ which further highlights the importance of this review. Other abnormalities including neuroma, oedema, and scarring were detected using MRI. These forms of BPBI fall into the category of post-ganglionic injury. Cases such as these still may require surgical treatment with nerve grafting so remain an important finding on MRI. These tests have implicit threshold.

The following factors relating to MRI acquisition were expected to have systematic differences: manufacturer, model, pulse sequences, field strength, postprocessing techniques, and display monitors. All images were reviewed by experienced radiologists.

### Prior tests

To check for signs of fractures, X-rays of the upper limbs may have been performed prior to MRI in some infants. Ultrasound and blood tests may have also been performed.

### Reference standard

Supraclavicular surgical exploration of the brachial plexus was the only reference standard for detecting root avulsion, pseudomeningocele, and any nerve abnormality, which was classed as perfect as the plexus is under direct visualisation. Surgical exploration may involve intraoperative tests such as somatosensory evoked potentials (SEPs) and bipolar nerve stimulation to help detect avulsion and these tests were included as part of the reference standard. The references standard had implicit threshold.

### Search strategy

On February 15, 2023, the following sources were searched from inception with no language restrictions:

- Embase
- PubMed
- Cochrane Central Register of Controlled Trials (CENTRAL)
- Google Scholar
- medRxiv and bioRxiv preprint archives

Forward and backward citation chasing of the reference lists of included studies was also performed using CitationChaser.^24^ EndNote was used for manual de-duplication.

### Study selection

Two review authors (JB and CH) independently screened titles and abstracts of identified citations using Rayyan.^25^ Full-text articles of the remaining records were independently assessed for eligibility against predefined criteria. Disagreements were resolved by discussion with review authors. At this stage, studies were included regardless of whether the reported data were fit for extraction and analysis. A PRIMSA flow diagram was used to record the study selection process and reasons for exclusion were noted. No specific threshold was used for the index and reference tests.

### Data extraction

Two review authors (JB and CH) independently extracted data. A copy of the datasheet is available at DOI 10.17605/OSF.IO/4AG3V. All authors of included papers were contacted to provide further data. Discrepancies were resolved by discussion with review authors.

### Assessment of methodological quality

The risk of bias and applicability of included studies were evaluated by JB using a tailored version of the Quality Assessment of Diagnostic Accuracy Studies (QUADAS)-2 tool^26^ (see Supplementary Materials).

### Data synthesis

ReviewManager 5^27^ was used to generate forest plots with estimates of sensitivity and specificity for all target conditions (root avulsion, pseudomeningocele, and any nerve abnormality) with both the patient and the nerve as the unit of analysis. Meta-analysis using MetaDTA^28^ was performed using a random effects bivariate binomial model for the primary target condition (root avulsion) with nerves as the unit of analysis. This was shown using a summary receiver operating characteristic (SROC) plot with the respective summary confidence and predictive regions overlaid. Due to limited data, a formal investigation of heterogeneity was not undertaken and nor was a sensitivity analysis. Root avulsion data with the patient as the unit of analysis and data regarding the secondary target conditions (pseudomeningoele and any nerve abnormality) were too sparse and heterogenous to conduct meta-analysis. Meta-analysis of proportions was performed in Stata v18^29^ using the meta suite. Confidence intervals (CI) were generated to the 95% level.

## Results

### Study selection

**Figure 1.**
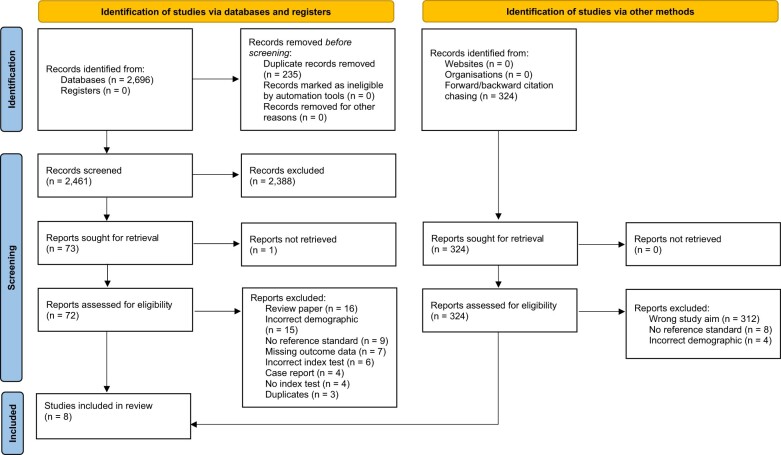
PRIMSA flow diagram.

In total, 2696 unique articles were identified which ultimately resulted in eight studies being included in the review (Figure 1).

### Study characteristics

The characteristics of included studies are presented in Table 1. All studies were performed between 1991 and 2019 and originated from the United States,^11^^,^^30–33^ Egypt,^10^ Poland,^34^ and Finland.^9^ Six studies were carried out prospectively, whereas two studies^30^^,^^32^ were of retrospective design. The median number of participants across all eight included studies was 15 (range 4-31). All corresponding authors were contacted to provide more detailed data but no responses were received.

**Table 1.** Study characteristics.

First author and year of publication	Recruitment time frame	Location	Study type	No. of patients	Mean patient age at scan (weeks)	Mean age at surgery (weeks)	Number of males	Patients with at least 1 root avulsion	Overall frequency of root avulsion	Reported outcomes
Abbott 2004	1997-2002	United States	Prospective cohort	15	Not described	130	6	0	0	PM, neuroma
Gad 2020	2016-2019	Egypt	Prospective cohort	15	63.4	Not described	10	Not described	19 (25)	Avulsion, neuroma, oedema
Gosk 2012	1998-2006	Poland	Prospective cohort	20	34.3	Not described	Not described	9	18 (19)	Avulsion
Grahn 2019	2007-2015	Finland	Prospective cohort	11	22.1	25.2	Not described	5	8 (15)	Avulsion, PM
Medina 2006	2001-2004	United States	Prospective cohort	31	29.2	Not described	19	Not described	Not described	PM, neuroma
Sherburn 1997	1991-1996	United States	Prospective cohort	14	Not described	54	Not described	5	Not described	Avulsion, PM
Smith 2008	2003-2006	United States	Case series	4	40	Not described	2	0	0	Avulsion, PM, neuroma
Somashekar 2014	Not described	United States	Prospective cohort	6	Not described	Not described	Not described	4	8 (27)	Avulsion, PM

Bracketed values are percentages.

Abbreviation: PM = pseudomeningocele.

In total, 116 infants were included (mean age 39 weeks). The overall proportion of males to females was difficult to determine given that in four studies^9^^,^^31^^,^^33^^,^^34^ data were extracted based on a smaller cohort of patients of which the number of males and/or females was not reported. Both the unit of analysis and target condition varied between studies. Six studies reported avulsion as a target condition,^9^^,^^10^^,^^31–34^ six reported pseudomeningocele,^9^^,^^11^^,^^30–33^ four reported neuroma,^10^^,^^11^^,^^30^^,^^32^ and one reported oedema.^10^ Four studies defined the nerve as the unit of analysis,^9^^,^^10^^,^^33^^,^^34^ with the remaining four reporting the patient as the unit of analysis.^11^^,^^30–32^ Five studies^9–11^^,^^32^^,^^34^ described the time from birth to imaging (mean time 38 weeks, standard deviation 14 weeks) and three^9^^,^^30^^,^^31^ reported the time from birth to surgery (median time 54 weeks).

Characteristics of the scanning protocols used are described in Table 2. A range of techniques were used including T1 and T2 weighted imaging, acquired using different techniques, and with variable use of fat suppression and diffusion-weighting. As image quality and interpretability depend heavily on sequences parameterisation, as well as patient-related factors such as motion, respiration, and blood flow, readers should defer to the methods of each paper for details of the sequences used.

**Table 2.** MRI sequences of included studies.

Study	MRI scanner	MRI sequences
Abbott 2004	GE (1.5 T)	Axial and coronal T1WI and T2WI
Gad 2020	Siemens Sempra (1.5 T)	T1WI and T2WI, DWIBS sequence scanning, 3D T2 STIR SPACE, and MR radial myelography
Gosk 2012	Marconi Medical Sytrunk (0.23 T)	Fast spin-echo 2D (FSE 2D)
Grahn 2019	Philips Achieva (1.5 T)	T1WI and T2WI spin-echo, T2 weighted BFFE
Medina 2006	Not stated	T1WI and T2WI spin-echo, STIR
Sherburn 1997	Not stated	Fast spin-echo 2D (FSE 2D)
Smith 2008	Not stated	Not stated
Somashekar 2014	Philips Ingenia (3 T)	3D T2 DRIVE

With the patient as the unit of analysis, operative exploration confirmed root avulsion in 23 out of 116 patients (pooled prevalence 27% [CI 6%, 54%]), pseudomeningocele associated with an ipsilateral root avulsion in 15% of patients (CI 0%, 47%) and any neural abnormality (avulsion, pseudomeningocele, or post-ganglionic injury) in 79% of patients (CI 52%, 98%). With the nerve as the unit of analysis, 53 out of 580 surgically explored spinal nerve roots were avulsed (pooled prevalence 11% [CI 2%, 24%]), 76 were abnormal (20% [CI 15%, 26%]) but no pseudomeningoceles were reported on a nerve-root basis. Studies were heterogenous in terms of the design, size, age of participants, scanning protocol, unit of analysis, and target condition.

### Risk of bias and applicability concerns

The risk of bias and applicability of each study are presented in Table 3. Seven studies^9^^,^^11^^,^^30–34^ were at risk of selection bias as it was unclear whether a consecutive or random sample of patients had been enrolled. MRI as an index test may have introduced a degree of bias in two studies^30^^,^^34^ as it was unclear whether the MRI results were interpreted without knowledge of the surgical exploration. In terms of the reference standard, surgical exploration could correctly classify root avulsion however the risk of bias was unclear in three studies^30^^,^^32^^,^^33^ because it was not stated that the surgeon was blinded to the results of MRI. However, as most studies performed were embedded into clinical practice it is unlikely that surgeons would be blinded to MRI results. There was a lack of detail reported across all studies relating to flow and timing, mainly concerning the interval between MRI and surgical exploration. Two studies^31^^,^^33^ were at a high risk of bias because not all participants were included in the analysis.

**Table 3.** Risk of bias and applicability concerns for each included study.

	Patient selection		Index test		Reference standard		Flow and timing
Study	Risk of bias	Applicability	Risk of bias	Applicability	Risk of bias	Applicability	Risk of bias
Abbott 2004	−	−	−	+	−	−	−
Gad 2020	−	−	+	−	+	+	−
Gosk 2012	−	−	−	+	+	x	−
Grahn 2019	+	−	+	+	+	+	−
Medina 2006	−	−	+	−	+	+	−
Sherburn 1997	−	−	+	−	+	−	x
Smith 2008	−	−	+	+	−	+	−
Somashekar 2014	−	−	+	−	−	+	x

Low (+), Unclear (−), High (x).

Applicability concerns in terms of patient selection were unclear in all eight studies due to factors such as prior tests, clinical scoring, description of the surgical procedure, and age at scanning and surgery not being described in adequate detail. Four articles^10^^,^^11^^,^^31^^,^^33^ were at unclear risk of applicability concerns relating to MRI as the index test because the images were interpreted by more than one radiologist which does not reflect clinical practice. In terms of surgical exploration as the reference standard, the concern for applicability was unclear in two studies^30^^,^^31^ because the criteria for diagnosis of root avulsion at surgery was incompletely described. The article by Gosk et al^34^ showed high concern as the results alone were reported without a description of the surgical procedure.

### Synthesis of results

**Figure 2.**
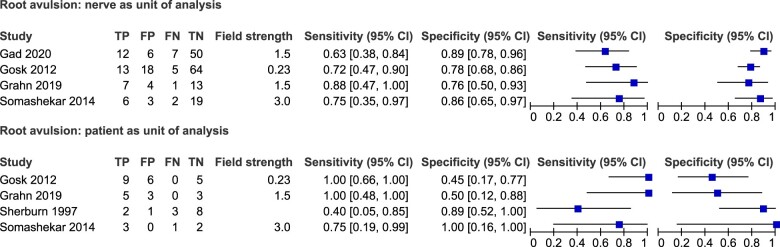
Forest plots of the sensitivity and specificity of MRI for root avulsion with associated 95% confidence intervals for the nerve (top) and patient (bottom) as the unit of analysis. TP = true positive, FN = false negative, FP = false positive, TN = true negative. Field strength measured in Tesla (T).

Forest plots of the sensitivity and specificity of MRI in detecting root avulsion with both the patient and nerve as the unit of analysis are presented in Figure 2. The mean sensitivity and mean specificity for MRI in detecting root avulsion with the nerve as the unit of analysis were 68% (CI 55%, 79%) and 89% (CI 78%, 95%), respectively. Field strength was not determined due to insufficient data. Meta-analysis was not performed on root avulsion with the patient as the unit of analysis due to limited data.

**Figure 3.**
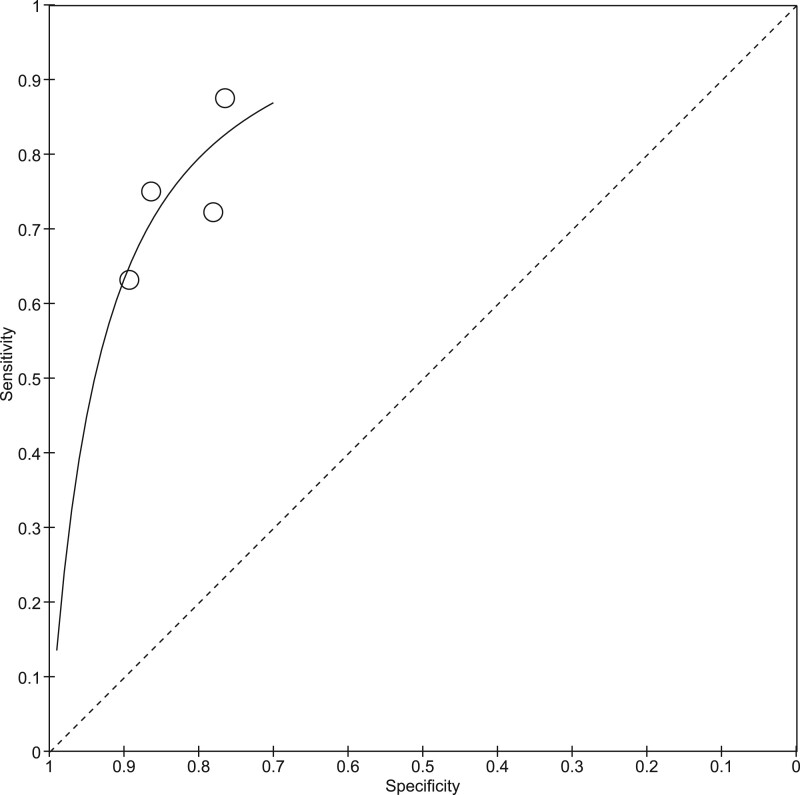
SROC plot showing the accuracy of MRI for detecting root avulsion with the nerve as the unit of analysis.

The accuracy of MRI for detecting root avulsion with the nerve as the unit of analysis is summarised by the SROC plot (Figure 3).

**Figure 4.**
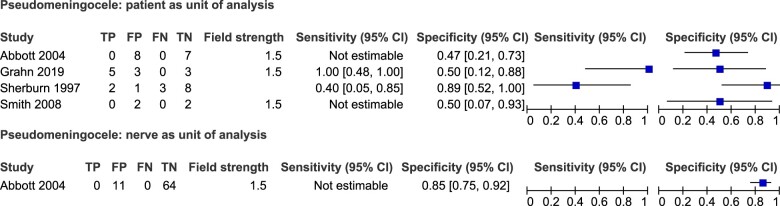
Forest plots of the sensitivity and specificity of MRI for pseudomeningocele as a surrogate marker of root avulsion with associated 95% confidence intervals for the patient (top) and nerve (bottom) as the unit of analysis. TP = true positive, FN = false negative, FP = false positive, TN = true negative. Field strength measured in Tesla (T).

The sensitivity and specificity of pseudomeningocele as a surrogate marker of root avulsion are presented as forest plots in Figure 4.

**Figure 5.**
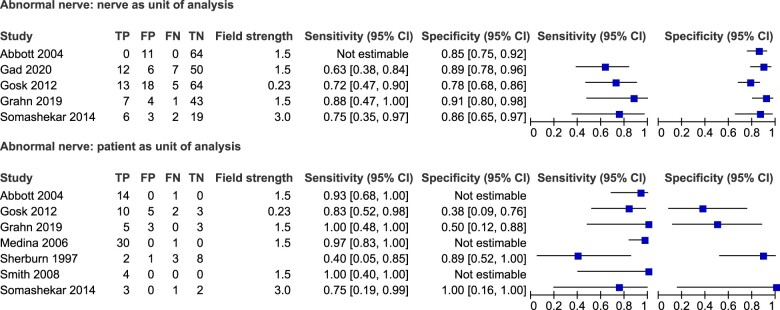
Forest plots of the sensitivity and specificity of MRI for any abnormal nerves with associated 95% confidence intervals for the nerve (top) and patient (bottom) as the unit of analysis. TP = true positive, FN = false negative, FP = false positive, TN = true negative. Field strength measured in Tesla (T).

The sensitivity and specificity of MRI for detecting any nerve abnormality are presented as forest plots in Figure 5.

## Discussion

In this evidence synthesis article, we show that morphological MRI has modest sensitivity (68%) and specificity (89%). In the real world, this would translate to 32 out of 100 avulsed nerves being missed and that MRI incorrectly classifies 11 in 100 normal nerves as avulsed. Ideally, the goal of MRI detection would be a sensitivity and specificity of 100% because results can determine surgical intervention in young babies. Sensitivity is arguably more important as a negative result could be fully relied upon to recommend avoidance of surgical exploration. Overall, this suggests that morphological MRI is currently not accurate enough and operative exploration should remain the diagnostic test of choice in children with BPBI.

Our results suggest pseudomeningocele is not a reliable marker of root avulsion as the sensitivity and specificity is highly variable between studies. The diagnostic accuracy of MRI for detecting any nerve abnormality in BPBI is also variable and it is not possible to confidently state that MRI alone can reliably diagnose nerve injury. Given the relatively high specificity of by-nerve detection, there is a role for MRI in pre-operative planning to help guide which nerves to explore and facilitate more efficient surgery, especially in relation to determining pre-/post-ganglionic injury.

Seven studies^10^^,^^11^^,^^30–34^ were potentially at risk of bias arising from patient selection which could have resulted in an unrepresentative sample of patients. It is therefore possible that the estimate of diagnostic accuracy is exaggerated.^35^ All eight studies showed some concern involving flow and timing which mainly related to a lack of clarity when describing the time points at which MRI and surgery were performed. Our criteria stated that the time between MRI and surgery should be less than 12 months. This is because abnormal findings, such as the presence of oedema, may have resolved by the time exploratory surgery is performed and thus erroneously inflate the false positive rate. Also, most institutions would explore the supraclavicular brachial plexus well before this time point which would limit the generalisability. Studies by Grahn et al^9^ and Medina et al^11^ reported this time period as 7 weeks and 4 weeks, respectively, however this was not stated by the remaining six articles. Overall, 57% of the QUADAS domains were classed as unclear or high risk of bias which is marginally higher than the average of 56% for reviews of diagnostic test accuracy.^36^ However, this assessment was conducted by a single author which is a limitation.

MRI may facilitate earlier treatment in those that require it and enable improved functional outcomes. However, no infants under 12 weeks of age were scanned so the feasibility of this approach remains unknown. Future studies involving younger cohorts of patients are required to determine the optimal age at which imaging should be performed. MRI protocols also varied across studies. For example, STIR techniques involve supressing signal from fat which allows nerves to be more easily visualised. Gad et al^10^ also incorporated diffusion-weighted sequences which enables characterisation of nerve microstructure and helps identify traumatic injury. In every case, it is vital the protocol is optimised to ensure both normal anatomy and pathology are clearly visualised. Given that our results demonstrate MRI to have modest accuracy in detecting root avulsion, development of novel protocols which improve anatomical visualisation and incorporate objective assessment of tissue function may help improve the overall accuracy of MRI. For example, recent hardware advances by manufacturers have enabled better imaging of peripheral nerves, such as: deep learning based imaging reconstruction (eg AIR Recon DL and Deep Resolve) which improves SNR, image resolution and fidelity and without a penalty,^37^ better conforming flexible neck and extremity coils which also enable parallel imaging and simultaneous multislice imaging to reduce scan times without compromising image quality.^38^^,^^39^ Similarly, advances in clinical imaging have shown that diffusion-tensor (and related q-space imaging techniques) yield objective, reliable, and repeatable measurements which are proxies of nerve health.^40^ The combination of these advances, as a supplement to current clinical imaging, is likely to bring about improvements in the diagnostic accuracy. We recommend a multicentre diagnostic accuracy study using fixed imaging parameters to better understand the topic.

Studies that reported the patient as the unit of analysis^11^^,^^30–32^^,^^34^ were prone to composite bias as the outcome was still recorded as positive irrespective of whether a single nerve root or all five roots were damaged. This made assessing the degree of severity on a per patient basis more difficult which is important clinically. Wade et al^16^ advocated using the nerve as the unit of analysis. Our evidence supports this principle as composite bias is avoided and a consistent approach to reporting brachial plexus injuries would allow larger amounts of data to be collated and result in more reliable estimates of diagnostic accuracy.

Given the potentially severe consequences of false negatives (permanent morbidity and loss of function) the threshold of acceptance for a procedure used to diagnose BPBI is high. Based on the findings of this study, which is the most comprehensive to date, MRI alone is not sufficiently accurate to detect nerve root avulsion in BPBI. Girard et al^41^ investigated the accuracy of MRI for root avulsion in BPBI, however they included single case reports and combined data comparing MRI to both surgery and clinical examination, which is known to only have moderate accuracy in diagnosing brachial plexus injury.^42^ Wade et al^16^ evaluated the accuracy of MRI in detecting root avulsion in traumatic adult cases. They also concluded that in adults, MRI offers modest diagnostic accuracy for root avulsion, with a mean sensitivity of 93% and mean specificity of 72%. The sensitivity appears to be lower in obstetric cases compared to the adult population, whereas the specificity is greater. Reasons for this difference may relate to the mechanism of injury. For example, sensitivity may be greater in adult cases because they usually arise from high impact collisions which are likely to result in extensive damage, whereas in BPBI the prolonged stretching forces experienced during labour reduce the likelihood of global avulsion.

In conclusion, surgical exploration should remain the diagnostic modality of choice for BPBI. Based on limited data, pseudomeningocele is not a reliable marker of root avulsion. Further research into imaging for BPBI is required to enable development of the high diagnostic accuracy required for the best management of this life altering condition.

## Supplementary Material

tqae214_Supplementary_Data
