# Supplementary material for: Diagnostic accuracy of MRI for detecting nerve injury in brachial plexus birth injury
Source: Br J Radiol. 2024 Oct 21;98(1165):36–44. doi: 10.1093/bjr/tqae214 (PMC11652713; doi:10.1093/bjr/tqae214)
Supplement: tqae214_Supplementary_Data [file tqae214_supplementary_data.zip › tqae214_Supplementary_Data/Supplementary material BJR.docx]

# Supplementary materials

## Search strategy

Embase

1. Exp Brachial Plexus Neuropathy/
2. Brachial Plexus/
3. (brachial adj1 plex*).tw.
4. (brachial and plex*).ti.
5. infant/
6. newborn/
7. Newborn disease/
8. Child/
9. Obstetric delivery/
10. Infant/
11. Infant*.ti.
12. Infant*.ab. /freq=2
13. Child*.ti.
14. Child*.ab. /freq=2
15. Obstetrics/
16. Obstetric*.ti.
17. Obstetric*.ab. /freq=2
18. Neonat*.ti.
19. Neonat*.ab. /freq=2
20. Perinatal*.ti.
21. Perinatal.ab. /freq=2
22. Obstetrical Brachial Plexus Palsy/
23. Neonatal brachial plexus palsy/
24. ((neonat* or perinatal*) adj2 brachial plexus pals*).tw.
25. Birth injury/
26. Birth injury.ti.
27. Birth injury.ab. /freq=2
28. Paralysis, Obstetric/
29. (obstetric pals* or birth pals*).tw,kw.
30. ((obstetric or birth or perinatal*) adj3 brachial plexus injur*).tw,kw.
31. (klumpke* pals* or klumpke* paralysis).tw,kw.
32. (erb* adj2 paralysis).tw,kw.
33. (erb* adj2 pals*).tw,kw.
34. Exp Nuclear Magnetic Resonance Imaging/
35. ((magnetic resonance adj (imag* or scan* or tomograph*) or MRI or MR imag* or MR tomograph* or MRT or NMR or NMRI or fMRI or chemical shift imag*)).tw.
36. (MR?).ti,ab.
37. (magnetic AND resonance).ti,ab.
38. (NMR).ti,ab.
39. (neurogra*).ti,ab.
40. (DTI).ti,ab.
41. ((diffusion and tensor) and imaging).ti,ab.
42. 1 or 2 or 3 or 4
43. 5 or 6 or 7 or 8 or 9 or 10 or 11 or 12 or 13 or 14 or 15 or 16 or 17 or 18 or 19 or 20 or 21
44. 42 and 43
45. 22 or 23 or 24 or 25 or 26 or 27 or 28 or 29 or 30 or 31 or 32 or 33
46. 34 or 35 or 36 or 37 or 38 or 39 or 40 or 41
47. 44 or 45
48. 46 and 47

PubMed

1. Exp Brachial Plexus Neuropathy/
2. Brachial Plexus/
3. brachial adj1 plex*
4. brachial and plex*
5. infant*/
6. newborn/
7. Newborn disease/
8. Child/
9. Obstetric delivery/
10. Obstetric*
11. Neonat*
12. Perinatal*
13. Obstetrical Brachial Plexus Palsy/
14. Neonatal brachial plexus palsy/
15. (neonat* or perinatal*) adj2 brachial plexus pals*
16. Birth injury/
17. obstetric pals* or birth pals*
18. (obstetric or birth or perinatal*) adj3 brachial plexus injur*
19. klumpke* pals* or klumpke* paralysis
20. erb’s adj2 paralysis
21. erb’s adj2 pals*
22. Exp Nuclear Magnetic Resonance Imaging/
23. (magnetic resonance adj (imag* or scan* or tomograph*) or MRI or MR imag* or MR tomograph* or MRT or NMR or NMRI or fMRI or chemical shift imag*)
24. MR?
25. magnetic AND resonance
26. NMR
27. neurogra*
28. DTI
29. (diffusion and tensor) and imaging
30. 1 or 2 or 3 or 4
31. 5 or 6 or 7 or 8 or 9 or 10 or 11 or 12
32. 30 and 31
33. 13 or 23 or 24 or 25 or 26 or 27 or 28 or 29 or 30 or 31 or 32 or 21
34. 22 or 35 or 36 or 37 or 38 or 39 or 40 or 29
35. 33 or 34
36. 34 and 35

Central

1. Brachial plexus
2. child OR obstetric OR infant OR neonat* OR perinat* OR erb* palsy OR klumpke* palsy

Google scholar (extracted first 5 pages of results from the following searches)

- “obstetric brachial plexus injury mri”
- “neonatal brachial plexus injury mri diagnostic accuracy”

## QUADAS-2

**QUADAS-2: Diagnostic Accuracy of Magnetic Resonance Imaging for Detecting Nerve Injury in Obstetric Brachial Plexus Injury**

**Study ID:**

Patients (setting, intended use of index test, presentation, prior testing):

Index test(s):

Reference standard and target condition:

**Domain 1: Patient Selection**

A. Risk of Bias

Describe the methods of patient selection:


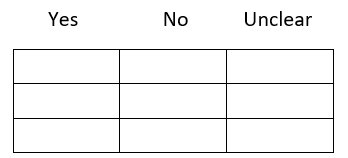


Was a consecutive or random sample of patients enrolled?
Was a case-control design avoided?
Did the study avoid inappropriate exclusions?

Could the selection of patients have introduced bias? **Low / High / Unclear***(Low = answered yes to all questions)
(High = answered no to at least one question)
(Otherwise, code as unclear)*

B. Concerns regarding applicability

Describe included patients (prior testing, presentation, intended use of index test and setting):

Is there concern that the included patients do not match the review question? **Low / High / Unclear***(Low = following factors were described and appropriate: prior tests, age at diagnosis, mode of delivery, clinical scoring, surgical method of exploration, field strength and pulse sequence(s) of MRI)
(Unclear = above factors were not described)
(High = multiple surgical explorations or MRIs were performed)*

**Domain 2: Index test**

A. Risk of bias

Describe the index test and how it was conducted and interpreted:


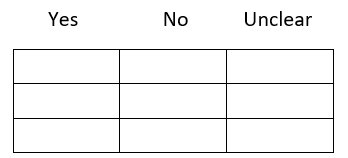
Were the MRI results interpreted without knowledge of the exploration?
If a threshold for either test was used, was it pre-specified?

Could the conduct or interpretation of the index test have introduced bias? **Low / High / Unclear**(Low = answered yes to both)
(High = answered no to both)
(Otherwise, code as unclear)

B. Concerns regarding applicability

Is there concern that the index test, its conduct, or interpretation **Low / High / Unclear**
differ from the review question?
*(Low = MRI images were interpreted by a single experienced radiologist and exploratory surgeries were performed by experienced surgeons)
(High = both tests were not performed by appropriate personnel)
(Otherwise code as unclear)*

**Domain 3: Reference standard**

A. Risk of bias

Describe the reference standard and how it was conducted and interpreted:


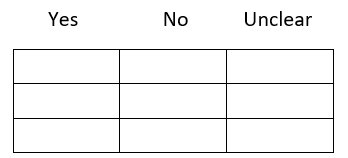


Is the reference standard likely to correctly classify the target condition?
Were the reference standard results interpreted without knowledge of the
results of the index test?

Could the reference standard, its conduct, or its **Low / High / Unclear**
interpretation have introduced bias?
*(Low = answered yes to both)
(High = answered no to both)
(Otherwise code as unclear)*

B. Concerns regarding applicability

Is there concern that the target condition as defined by the **Low / High / Unclear**
reference standard does not match the review question?
*(Low = criteria for root avulsion at surgery was clearly defined and results are reported)*
*(High = no description of the reference standard in the methods and the results alone are reported)
(Unclear = criteria for diagnosis of root avulsion at surgery was unclear or incompletely described in the methods and results are reported)*

**Domain 4: Flow and timing**

A. Risk of bias

Describe any patients who did not receive a preoperative MRI and/or exploration or who were excluded from the 2x2 table:

Describe the time interval and any interventions between index test(s) and reference standard:


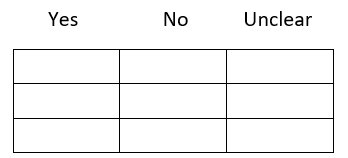


Was the interval between MRI and exploration <12 months?
Did all cases receive the same brachial plexus exploration?
Were all patients included in the analysis?

Could the patient flow have introduced bias **Low / High / Unclear**
*(Low = answered yes to all questions)
(High = answered no to at least one question)
(Otherwise code as unclear)*
